# Fast and efficient three-step target-specific curing of a virulence plasmid in *Salmonella enterica*

**DOI:** 10.1186/s13568-015-0139-y

**Published:** 2015-08-14

**Authors:** Marcos H de Moraes, Max Teplitski

**Affiliations:** Department of Soil and Water Science, University of Florida/IFAS, Gainesville, FL 32611 USA; Genetics Institute Rm 304, Cancer and Genetics Research Center, University of Florida, Gainesville, FL 32611 USA

**Keywords:** Virulence plasmid, *Salmonella enterica* sv. Typhimurium, I-*Sec*I nuclease, λ-Red mutagenesis

## Abstract

Virulence plasmids borne by serovars of *Salmonella enterica* carry genes involved in its pathogenicity, as well as other functions. Characterization of phenotypes associated with virulence plasmids requires a system for efficiently curing strains of their virulence plasmids. Here, we developed a 3-step protocol for targeted curing of virulence plasmids. The protocol involves insertion of an I-*Sec*I restriction site linked to an antibiotic resistance gene into the target plasmid using λ-Red mutagenesis, followed by the transformation with a temperature-sensitive auxiliary plasmid which carries I-*Sec*I nuclease expressed from a tetracycline-inducible promoter. Finally, the auxiliary plasmid is removed by incubation at 42 °C and the plasmid-less strains are verified on antibiotic-containing media. This method is fast and very efficient: over 90 % of recovered colonies lacked their virulence plasmid.

## Introduction

In many pathogens, including *Salmonella enterica*, virulence determinants are often carried on plasmids (rev. Etcheverria and Padola 2013; Fabrega and Vila 2013). Virulence plasmids are found in *Salmonella enterica* serovars Abortusovis, Choleraesuis, Dublin, Enteritidis, Gallinarum/Pullorum, Paratyphi C, Sendai, and Typhimurium (Akiba et al. 1999; Barrow and Lovell 1988; Barrow et al. 1987; Danbara et al. 1992; Gulig and Curtiss 1987; Libby et al. 1997; Liu et al. 2009; Nakamura et al. 1985; Ou et al. 1990), all of which are of medical or veterinary concern. These plasmids function as virulence determinants, contributing to pathogenesis of *S. enterica*, especially during the systemic phase of the disease (Libby et al. 1997; Uzzau et al. 2000). Virulence plasmids vary in size, ranging from 50 kb (sv. Choleraesuis) to 285 kb (sv. Sendai) (Feng et al. 2012). Despite overall differences in composition, some genetic components are conserved, most notably the virulence determinant *spv* operon which is expressed during infection and codes for SpvB, a toxin that ADP-ribosylates actin of the macrophage cell destabilizing its cytoskeleton (Heithoff et al. 1997; Lesnick et al. 2001; Tezcan-Merdol et al. 2001) and SpvC, a phosphothreonine lyase that helps subvert natural inflammation response to *Salmonella enterica* (Haneda et al. 2012; Mazurkiewicz et al. 2008). Intra-serovar diversity in plasmid composition has also been reported, although, on a more limited scale. For example, insertions led to the acquisition of antibiotic resistance genes in the virulence plasmid of the serovar Typhimurium, thus making it not only a virulence determinant, but also the source a multi-drug resistance phenotype (Herrero-Fresno et al. 2015).

Defining functions of virulence plasmids requires a process known as “plasmid curing”. Different methods were developed to cure bacteria of their virulence plasmids, they involve heat or chemical treatments to exclude the plasmid from the cell. Substances with DNA intercalating properties, gyrase inhibiting effects and selections against phenotypes associated with the plasmid were successfully employed in different bacterial species and plasmids [rev. (Trevors 1986)]. However, these methods have some important caveats: treatment with harsh chemicals that affect properties of DNA or functions of DNA replicating machine tend to select for mutants (although this effect has not been exhaustively explored). Counter-selection, using sensitivity to sucrose or fusaric acid (encoded by *sacB* or *tetRA*, respectively) can be fastidious and time-consuming, with low efficiency.

An alternative to methods based on chemical or temperature stresses is the curing by incompatibility. To this end, the host bacterium is transformed with a second plasmid from the same incompatibility group, this plasmid interferes with the replication of the native plasmid by competing for the replication and partitioning systems or interfering with the ability of the plasmid to correct fluctuations in its copy number (Novick 1987; Tinge and Curtiss 1990). To increase the potential of interfering with the resident plasmid, the secondary plasmid is usually smaller, high copy and carries an antibiotic marker to help the process with selective pressure. Despite the advantages, this system also requires the same counter-selective methods to remove the secondary plasmid from the host.

In this paper we describe a new system to cure the virulence plasmid of *Salmonella enterica* that is based on the λ-Red recombination and I-*Sec*I meganuclease. This method differs from all other approaches because it is not based on exposure to stress or the use of incompatibility plasmids. It does not require fastidious counter-selection and works with high efficiency: over 90 % of the recovered colonies were cured of the virulence plasmid.

## Materials and methods

### Bacterial strains and culture conditions

Bacterial strains and plasmids used in this study are listed in Table 1. *S. enterica* and *Escherichia coli* strains were propagated in LB (Luria–Bertani) broth (Fisher Scientific) at 37 °C unless otherwise specified in this text. As appropriate, media were supplemented with 100 µg/ml ampicillin or 20 µg/ml chloramphenicol.

**Table 1:** Strains used in the study

Strains	Characteristic	References
*S. enterica* sv. Typhimurium ATCC 14028	Wild type	ATCC
*S. enterica* sv. Typhimurium MHM21	*spvA*::*cat*, I-*Sec*I recognition site	This study
*S. enterica* sv. Typhimurium MHM113	Lacks virulence plasmid p14028	This study
Plasmids		
pWRG100	*cat*, I-*Sec*I recognition site	(Blank et al. 2011)
pWRG99	λ-Red genes under control of arabinose inducible promoter (P_*BAD*_), I-*Sec*I endonuclease under control of tetracycline inducible promoter (P_*tetA*_), temperature sensitive, Amp^R^	(Blank et al. 2011)
pMHM199	I-*Sec*I endonuclease under control of tetracycline inducible promoter (P_*tetA*_), temperature sensitive, Amp^R^	This study

The effect of different concentrations of anhydrotetracycline (AHT) on bacterial growth was analyzed by growth curves of *S. enterica* sv. Typhimurium MHM21 pMHM199. Overnight cultures were adjusted to OD_600_ = 0.01 in LB medium with 100 µg/ml ampicillin, and 200 µl of the culture were transferred to a well of a 96-wells plate with a range of AHT concentrations (0, 125, 500 and 1,000 ng/ml), with each condition assayed in triplicates. The plate was incubated in a plate reader (Victor^3^, Perkinelmer) at 30 °C and the absorbance at 590 nm was measured hourly.

### Cloning and strains construction

Plasmid pMHM199 was constructed by amplifying I-*Sec*I gene under the tetracycline inducible promoter P_*tetA*_, the regulator *tetR*, and the ampicillin resistance gene as well as the oriR101 origin of replication and temperature-sensitive repA101ts with primers CGAAATCCACTGAAAGCACA and AAGCTGCTTTTGAGCACCAC using pWRG99 (Blank et al. 2011) as a template and Q5^®^ High-Fidelity DNA Polymerase (New England Labs). The resulting amplicon was treated with T4 DNA Ligase (New England Biolabs) under the conditions that favor intra-molecular ligation. The resulting plasmid was confirmed by the restriction digest. pMHM199 carries the endonuclease I-*Sec*I under the promoter responsive to anhydrotetracycline P_*tetA*_.

The cassette containing the I-*Sec*I site and chloramphenicol resistance gene was amplified from the template pWRG100 with primers (MHM91 *ATGAATATGAATCAGACCACCAGTCCGGCACTTTCACAGGTCGAAACCGC***CGCCTTACGCCCCGCCCTGC** and MHM92 *CTAAACTGCCGGCTGGCACGCAGAGTACCCGCAATCAACTGTTCCACCTG***CTAGACTATATTACCCTGTT**, where boldface letters indicate regions of homology with pWRG100 and italicized letters indicate homology with the *spvA* gene) using OneTaq^®^ DNA polymerase. The resulting PCR fragment was inserted in the place of the *spvA* gene by λ-Red recombination (Datsenko and Wanner 2000). Briefly, electrocompetent cells were prepared using *Salmonella enterica* sv. Typhimurium 14028 harboring pKD46 grown in LB broth at 30 °C supplemented with 100 µg/ml ampicillin and 10 mM l-arabinose until OD_600_ = 0.6–0.8, for approximately 3–4 h.

The *spvA* PCR cassettes (10–50 ng) were electroporated into 50 µl aliquots of electrocompetents cells. Electroporations were carried out using MicroPulser (BioRad) using manufacturer’s instructions. Cells were recovered in SOB (Super Optimal Broth) containing 2 % w/v tryptone, 0.5 % w/v yeast extract, 10 mM NaCl, 10 mM MgCl_2_, 2.5 mM KCl (Sambrook and Russell 2001) and plated onto LB agar with 20 µg/ml chloramphenicol. The insertion was transduced into *S. enterica* sv. Typhimurium 14028 by phage P22. All insertions were confirmed by PCR (MHM29 CAGAATGGTCTGCTGCTCAC and MHM30 GGATTTCATTCTGTTTATTTTCAGG).

### Efficiency of the plasmid curing system

Approximately 100 ng of purified pMHM199 were added to 50 µl of electrocompetent cells prepared from MHM21 strain. The cells were electroporated in 0.2 cm gap cuvettes with a MicroPulser (BioRad). Cells were resuspended in 1 ml of SOB medium and recovered for 1 h at 30 °C. The cells were divided in 350 µl aliquots and plated onto LB agar with 100 µg/ml ampicillin and supplemented with 0, 125 or 500 ng/ml AHT. The plates were incubated at 30 °C overnight. After the incubation, 50 colonies from each treatment were patched onto a new LB agar 100 µg/ml ampicillin plate and incubated at 30 °C overnight to purify the colonies and ensure that only bacteria carrying pMHM199 were present. The colonies were transferred to a new LB agar 20 µg/ml chloramphenicol plate and incubated at 37 °C overnight. The colonies that grew showing chloramphenicol resistance were defined as carrying pLST and the colonies that were chloramphenicol sensitive were defined as cured from pLST. To confirm the curing of pLST, the DNA from three chloramphenicol sensitive colonies and MHM21 as control were extracted using GenElute Bacterial DNA kit (Sigma-Aldrich) for a PCR targeting the plasmid genes *repA* (MHM1 GGCTATAGAGTGCGGTCTGG and MHM2 TCCGTGCAGTTTACGTTCAG, *spvA* (MHM29 and MHM30) and the chromosomal gene *phoN* (MHM7 TCATTTGCTGTGGCCAGTT and MHM8 TTATTGCCTGATCCGGAGTG) as a control.

## Results

### Plasmid curing

**Fig. 1:**
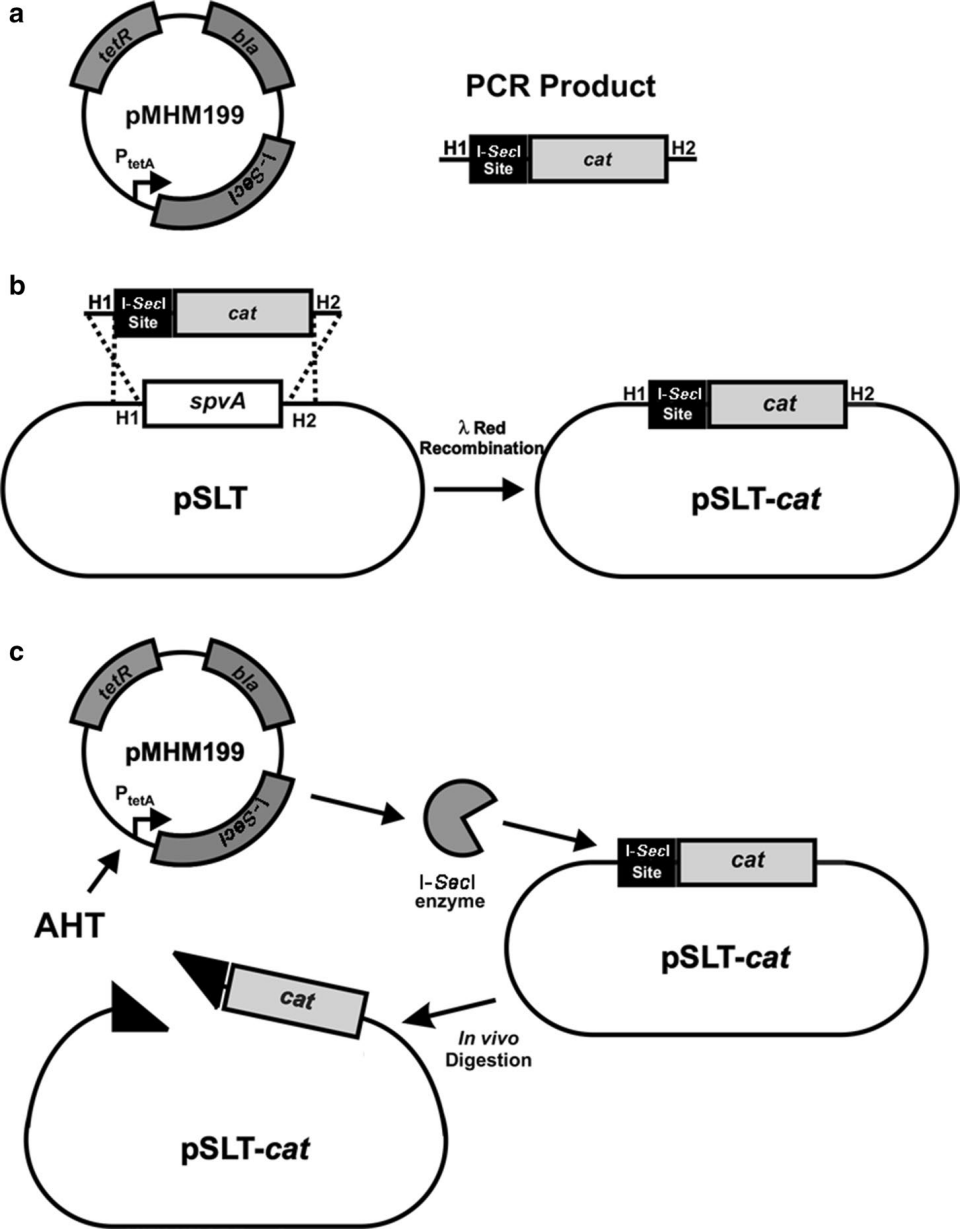
Steps involved in plasmid curing. **a** Plasmid pMHM199 contains I-SecI restriction endonuclease gene under the tetracycline inducible promoter P_tetA_, the regulator *tetR*, and the ampicillin resistance gene as well as the oriR101 origin of replication and temperature-sensitive repA101ts. **b** Deletion cassette is built by PCR using primers ATGAATATGAATCAGACCACCAGTCCGGCACTTTCACAGGTCGAAACCGC**CGCCTTACGCCCCGCCCTGC** and CTAAACTGCCGGCTGGCACGCAGAGTACCCGCAATCAACTGTTCCACCTG**CTAGACTATATTACCCTGTT** and plasmid pWRG100 as a template [*in bold*, regions of homology with pWRG100 (Blank et al. 2011)]. The cassette contains the I-*Sec*I site adjacent to the chloramphenicol resistance gene *cat*, flanked by the homology regions to *spvA* gene (H1 and H2). **c** λ Red recombination step. The homology regions on the insertion cassette recombine with the *spvA* plasmid gene by λ Red recombination, excluding the *spvA* and substituting it with the I-*Sec*I site linked to the *cat* gene generating the strain MHM21. **d** In vivo digestion. The plasmid pMHM199 is electroporated into MHM21, which is then plated onto LB agar containing ampicillin to select the positive transformants, and anhydrotetracycline.

The procedure for the technique is shown in Fig. 1. The first step (Fig. 1a) requires PCR amplification of the I-*Sec*I site and a chloramphenicol resistance gene (*cat*) using pWRG100 as a template. The PCR primers included regions of homology to pWRG100 CGCCTTACGCCCCGCCCTGC and CTAGACTATATTACCCTGTT on the 3′ ends (Blank et al. 2011). The PCR product replaced *spvA* gene of pSLT using λ-Red recombination (Datsenko and Wanner 2000). The resulting strain (MHM21 pSLT-*cat*) was confirmed to be resistant to chloramphenicol and the presence of the I-*Sec*I site was confirmed by PCR (Fig. 1b).

The next step (Fig. 1c) consisted of the electroporation of MHM21 pSLT-*cat* with the helper plasmid pMHM199, which carries the I-*Sec*I endonuclease gene under the tetracycline-activated promoter P_*tetA*_ and *tetR*. The transformation reaction was directly plated onto LB agar containing ampicillin 100 μg/ml to maintain pMHM199 and AHT that induces the expression of I-*Sec*I (via TetR). Then, the I-*Sec*I endonuclease targets the I-*Sec*I site on the 5′ end of the *cat* gene, which linearizes the virulence plasmid, leading to its loss in the subsequent generations.

The pMHM199 is a low copy, temperature-sensitive plasmid with replication origin based on pSC101, which allows easy curing by growing the cells in 42 °C for 6 h. The last step consists in the selection and confirmation of colonies cured of pSLT, by first selecting colonies that were chloramphenicol sensitive followed by PCR targeting the plasmid genes *spvA* and *repA*. The use of two targets at distant position on the plasmid helps to avoid the selection of mutants with segment deletions and integration to the chromosome. The resultant colonies were also confirmed to be ampicillin-sensitive, indicating loss of pMHM199.

### Effect of AHT on cell growth

**Fig. 2:**
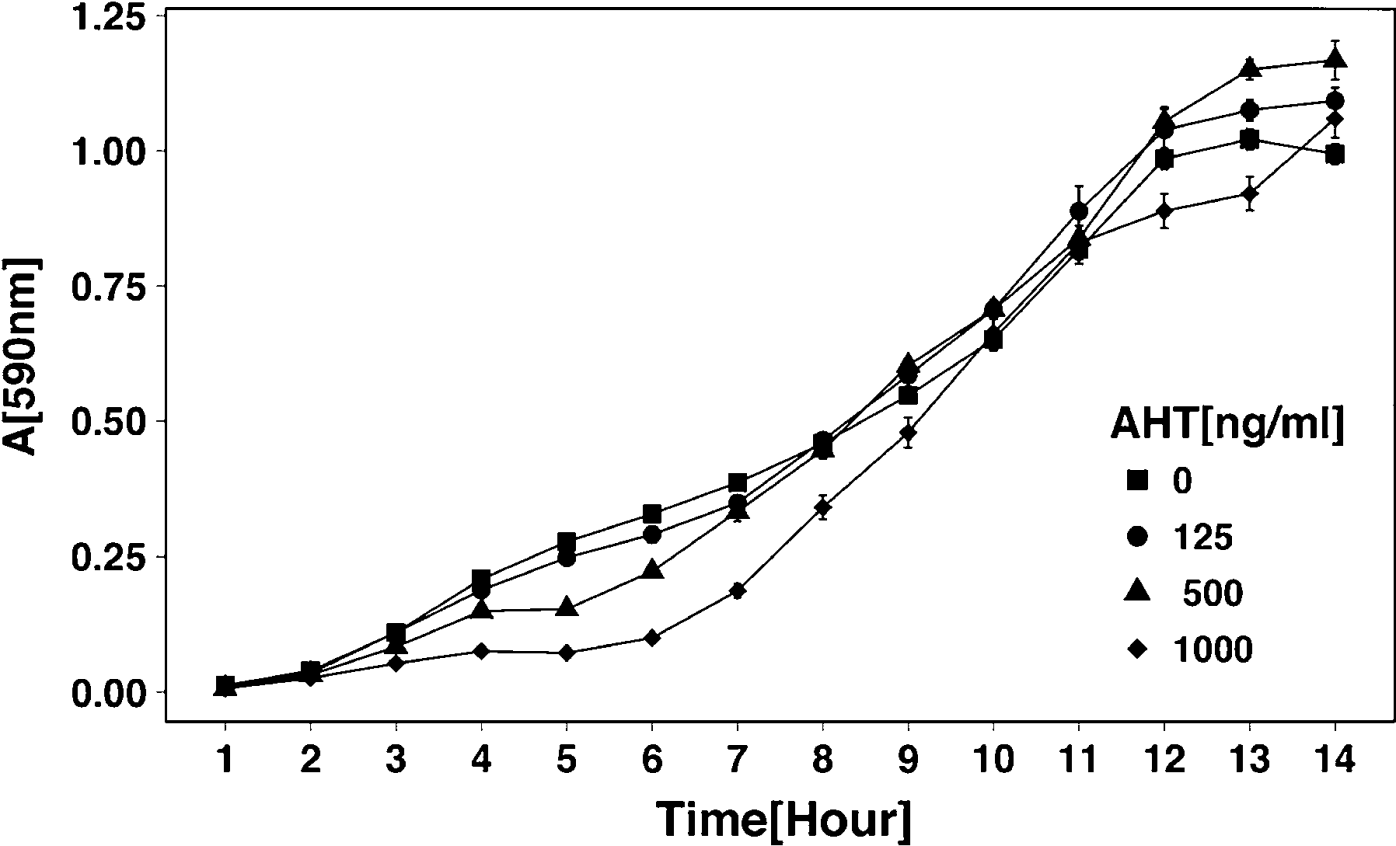
Growth of MHM21 in LB medium supplemented with different amounts of anydrotetracycline. Overnight cultures of MHM21 (*spvA*::[I-*Sec*I site, *cat*]) were adjusted to OD_600_ = 0.01 in LB medium with 100 µg/ml ampicillin. Two hundred microliters of the culture were transferred to wells of a 96-well plate with different concentrations of AHT (0, 125, 500 and 1,000 ng/ml) with each condition assayed in triplicates. The plate was incubated in a plate reader (Victor^3^, Perkin Elmer) at 30 °C and the absorbance at 590 nm was measured hourly.

Anhydrotetracycline (AHT) is a tetracycline derivative that has a higher affinity for TetR, allowing the chemical to be used as an inducer in vivo. However, AHT also exhibits bacteriostatic effects. To determine at which concentrations AHT is most effective as an inducer and less toxic to the cells, a growth curve with the strain MHM21 pMHM199 was constructed with a gradient of AHT concentrations (0, 125, 500, 1,000 ng/ml), as shown in Fig. 2. MHM21 pMHM199 was able to grow under all conditions, but growth inhibition was observed under the the higher concentrations (500, 1,000 ng/ml) while growth in media containing less than 125 ng/ml remained close to the negative control. Because of the detrimental effects of concentration 1,000 ng/ml, it was exclude from further experiments to test the system efficiency.

### Plasmid curing efficiency

**Fig. 3:**
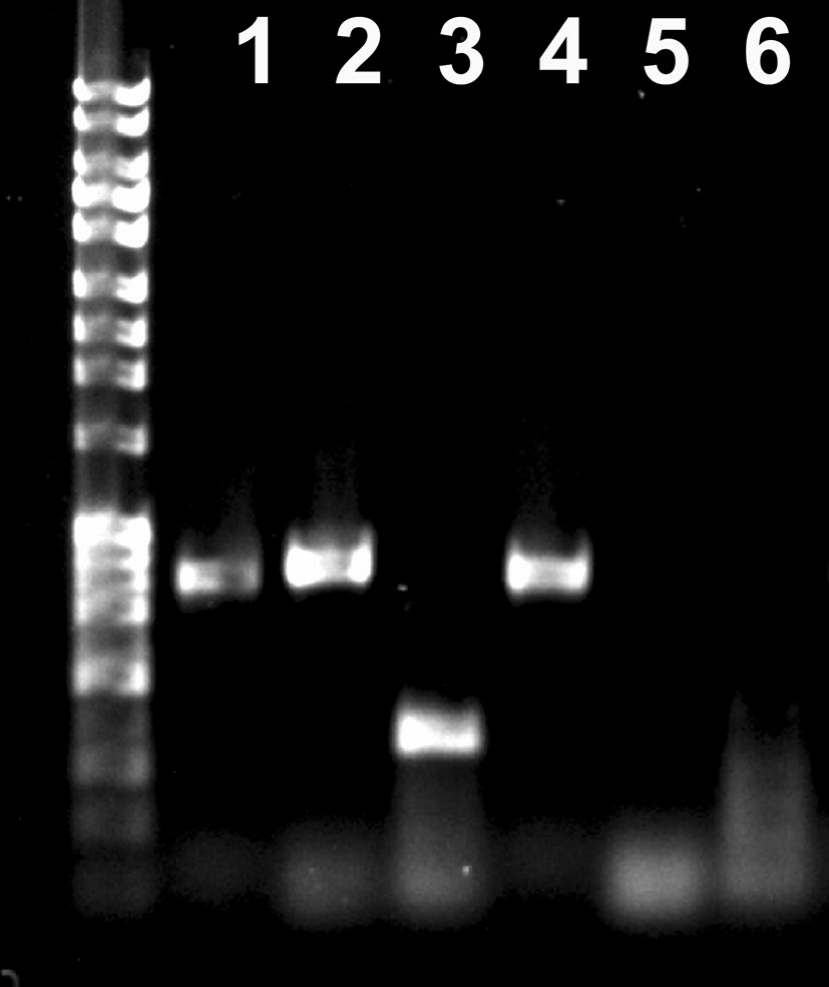
Confirmation of the loss of pSLT. Agarose gel (0.9 %) with PCR products to confirm curing of virulence plasmid. *Lanes 1*, *2* and *3* are reactions performed with strain MHM21 before plasmid curing; the strain cured of pSLT was used as a template in *lanes 4*, *5* and *6*. *Lanes 1* and *4* contain PCR products targeting chromosomal gene *phoN* (amplified using TCATTTGCTGTGGCCAGTT and TTATTGCCTGATCCGGAGTG; *lanes* *2* and *5* contain PCR product stargeting *spvA*::[I-*Sec*I site, *cat*] with CAGAATGGTCTGCTGCTCAC and GGATTTCATTCTGTTTATTTTCAGG; and *lanes* *3* and *6* contain PCR products targeting plasmid gene *repA* (with primers GGCTATAGAGTGCGGTCTGG and TCCGTGCAGTTTACGTTCAG).

After the strain MHM21 pSLT-*cat* was transformed with pMHM199, the cells were plated onto LB agar containing a range of AHT concentrations (0, 125, 500 ng/ml) to determine the efficiency and best condition for the plasmid curing method. To quantify the loss of virulence plasmid, colonies were tested for chloramphenicol resistance, and the loss of resistance was interpreted as loss of the plasmid. To confirm that the chloramphenicol-susceptible colonies did not harbor virulence plasmid, a PCR targeting plasmid genes *spvA* and *repA* and chromosomal gene *phoN* as a positive control was carried out. As expected all colonies were chloramphenicol resistant with the absence of AHT, and a surprisingly high number of colonies were sensitive to chloramphenicol after growing under AHT, 90 % for 125 ng/ml and 93 % for 500 ng/ml. The loss of resistance was very similar for each treatment, indicating that even under the lower tested AHT concentration the system was successful in curing the virulence plasmid. PCR amplification of *spvA* and *repA* failed in all chloramphenicol-susceptible colonies, indicating that all chloramphenicol-sensitive isolates lost pSLT-*cat* (Fig. 3).

## Discussion

Plasmids carry functions important for interactions of pathogenic and symbiotic bacteria with their hosts. In *S. enterica*, virulence plasmids encode functions involved in virulence (such as *spv*), contributing to pathogenesis of *S. enterica*, especially during the systemic phase of the disease (Libby et al. 1997; Uzzau et al. 2000). Elucidation of the phenotypes associated with virulence plasmids required their efficient curing from the bacterial cells.

Various methods have been used to generate plasmid-less strains of *S. enterica*, however, most of them either have low efficiency or require that bacteria are cultured successively under the stressful conditions that likely result in the accumulation of second-site mutations. The method for curing virulence plasmids reported here is over 90 % efficient, which is significantly higher than 10^−6^–10^−7^ per generation reported for the same plasmid using novobiocin (Gulig and Curtiss 1987) or using the incompatibility plasmid and destabilization of the *par* locus (Tinge and Curtiss 1990). The latter method was 82 % efficient, however, 10 generations were required to achieve this level of efficiency (Tinge and Curtiss 1990).

The method described here could also be particularly useful in curing individual plasmids from strains that carry multiple plasmids. For example, multi-drug resistant strains of *S. enterica* currently circulating in Europe (Garcia et al. 2014) contain multiple plasmids, and the method described here could be used to efficiently remove individual plasmids. This method could also be used for the generation of vaccine strains, similar to the study of Matsui et al. (2015) who demonstrated that plasmid-cured *S. enterica* strains used as live vaccines have been tested as live vaccine strains providing nearly complete protection against virulent *S. enterica* in mice (Matsui et al. 2015).
